# Evaluating and addressing demographic disparities in medical large language models: a systematic review

**DOI:** 10.1186/s12939-025-02419-0

**Published:** 2025-02-26

**Authors:** Mahmud Omar, Vera Sorin, Reem Agbareia, Donald U. Apakama, Ali Soroush, Ankit Sakhuja, Robert Freeman, Carol R. Horowitz, Lynne D. Richardson, Girish N. Nadkarni, Eyal Klang

**Affiliations:** 1 The Division of Data-Driven and Digital Medicine (D3M), Icahn School of Medicine at Mount Sinai, New York, NY USA; 2 Diagnostic Radiology, Mayo Clinic, Rochester, MN USA; 3 Ophthalmology Department, Hadassah Medical Center, Jerusalem, Israel; 4 The Charles Bronfman Institute of Personalized Medicine, Icahn School of Medicine at Mount Sinai, New York, NY USA; 5 Institute for Health Equity Research, Icahn School of Medicine at Mount Sinai, New York, NY USA

## Abstract

**Background:**

Large language models are increasingly evaluated for use in healthcare. However, concerns about their impact on disparities persist. This study reviews current research on demographic biases in large language models to identify prevalent bias types, assess measurement methods, and evaluate mitigation strategies.

**Methods:**

We conducted a systematic review, searching publications from January 2018 to July 2024 across five databases. We included peer-reviewed studies evaluating demographic biases in large language models, focusing on gender, race, ethnicity, age, and other factors. Study quality was assessed using the Joanna Briggs Institute Critical Appraisal Tools.

**Results:**

Our review included 24 studies. Of these, 22 (91.7%) identified biases. Gender bias was the most prevalent, reported in 15 of 16 studies (93.7%). Racial or ethnic biases were observed in 10 of 11 studies (90.9%). Only two studies found minimal or no bias in certain contexts. Mitigation strategies mainly included prompt engineering, with varying effectiveness. However, these findings are tempered by a potential publication bias, as studies with negative results are less frequently published.

**Conclusion:**

Biases are observed in large language models across various medical domains. While bias detection is improving, effective mitigation strategies are still developing. As LLMs increasingly influence critical decisions, addressing these biases and their resultant disparities is essential for ensuring fair artificial intelligence systems. Future research should focus on a wider range of demographic factors, intersectional analyses, and non-Western cultural contexts.

**Graphic Abstract:**

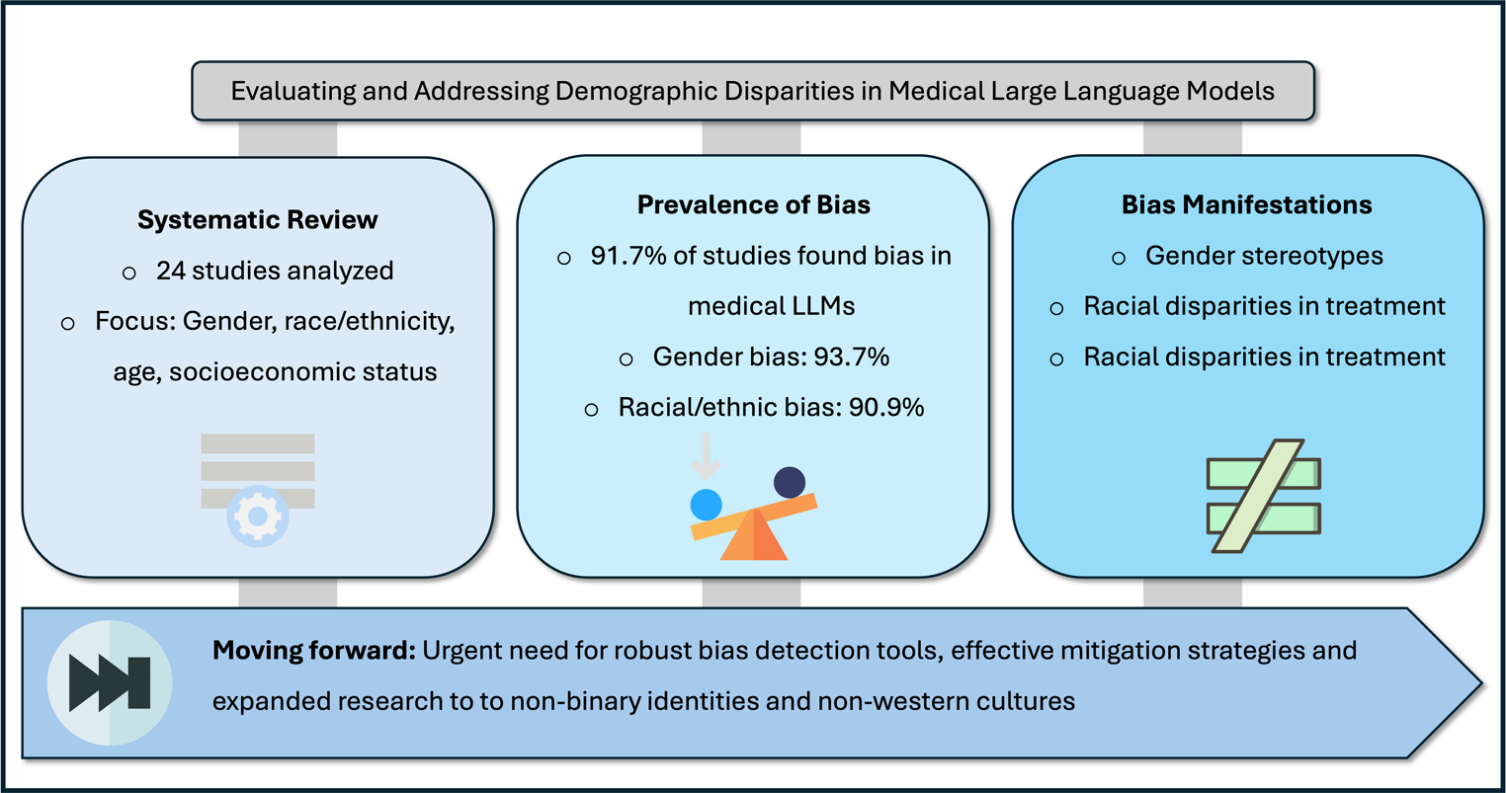

**Supplementary Information:**

The online version contains supplementary material available at 10.1186/s12939-025-02419-0.

## Introduction

LLMs are being integrated in multiple sectors, including healthcare [1, 2]. These models, however, are trained on human-generated text, which often contains biases [3–5]. The extent and nature of demographic biases in LLMs remain under-researched. Some studies reveal concerning examples, such as LLMs being less likely to recommend advanced imaging for patients from underrepresented racial groups [6]. Similar biases have been observed in legal and other professional domains [7]. These biases, which could be influenced by factors like model architecture, training data, and deployment context, can impact critical decisions and have potentially severe consequences [4].

Recent research has shown that commercially available LLMs can perpetuate race-based medical misconceptions [3, 5, 6]. In a study evaluating four LLMs across multiple healthcare scenarios, all models demonstrated instances of promoting debunked racial stereotypes in medicine [8]. This can be challenging to detect and measure. Current mitigation strategies include debiasing algorithms, prompt engineering, and diverse training data [9]. However, the rapid evolution of these models necessitates ongoing research to ensure future developments promote fairness. This is important given that a recent survey of FDA-approved AI clinical decision support tools found none included a bias evaluation, defined as an analysis to determine whether the tool’s outcomes are fair across different patient groups [10].

LLMs are increasingly used in healthcare. However, these models maybe trained on data that often include human biases, which may lead to unequal outcomes in clinical practice. Although some studies have identified these possible biases, a clear synthesis of the evidence is missing.

We systematically reviewed current research on demographic biases in medical LLMs, aiming to identify prevalent bias types, assess measurement methods, and evaluate mitigation strategies.

## Materials and methods

### Registration and protocol

We conducted a systematic review following the Preferred Reporting Items for Systematic Reviews and Meta-Analyses (PRISMA) guidelines [11]. The protocol was registered with the International Prospective Register of Systematic Reviews (PROSPERO), registration number: CRD42024578467 [12].

### Data sources and search strategy

We searched PubMed, Embase, Web of Science, APA PsycInfo, and Scopus for studies published between January 1, 2018, and July 31, 2024. The search strategy combined terms related to LLMs (e.g., “LLM”, “GPT”, “BERT”) with terms for bias and fairness. We validated our search strings through iterative testing and refinement. We supplemented database searches with manual screening of reference lists from included studies. The full search strategy is available in the Supplements. We developed our search strategy following the methods outlined in Chap. 4 of the Cochrane Handbook for Systematic Reviews of Interventions (version 6.4) [13]. We used the Rayyan web application for initial screening [14].

### Study selection

Two reviewers independently screened titles and abstracts using Rayyan software (MO, EK). We obtained full-text articles for all potentially eligible studies. The two reviewers then independently assessed these articles for inclusion. Disagreements were resolved by discussion or arbitration by a third reviewer. The full process is detailed the Supplements.

We included peer-reviewed studies that evaluated demographic biases in LLMs applied to medical or healthcare tasks. We defined demographic bias as systematic variation in model outputs based on characteristics such as gender, race, or age [15]. We excluded studies of non-LLM models, those focusing solely on model performance without addressing bias, and non-peer-reviewed materials.

### Data extraction and quality assessment

We developed a standardized form for data extraction. One reviewer extracted data, which was verified by a second reviewer. We extracted information on study design, LLM type, types of bias, measurement methods, and key findings. The full process is detailed the Supplements.

We assessed study quality using a multi-approach method with the JBI Critical Appraisal Checklist for Diagnostic Test Accuracy Studies and the JBI Critical Appraisal Checklist for Analytical Cross-Sectional Studies. These tools offers a structured framework that can be adapted to assess LLM bias studies, which often share methodological similarities with diagnostic accuracy research. Both fields evaluate outputs against expected standards, examine rates of incorrect classifications, and frequently involve classification tasks. Given the current lack of specific quality assessment tools for LLM bias studies, the JBI checklist provides a flexible approach that can be modified to evaluate crucial aspects such as data selection, bias measurement methods, and control of confounding factors in LLM research.

### Data synthesis and analysis

Due to the heterogeneity of included studies, we conducted a narrative synthesis. We categorized studies by type of bias examined, measurement approach, and mitigation strategies proposed. Where possible, we presented quantitative summaries of bias measurements across studies.

## Results

### Search results and study selection

**Fig. 1:**
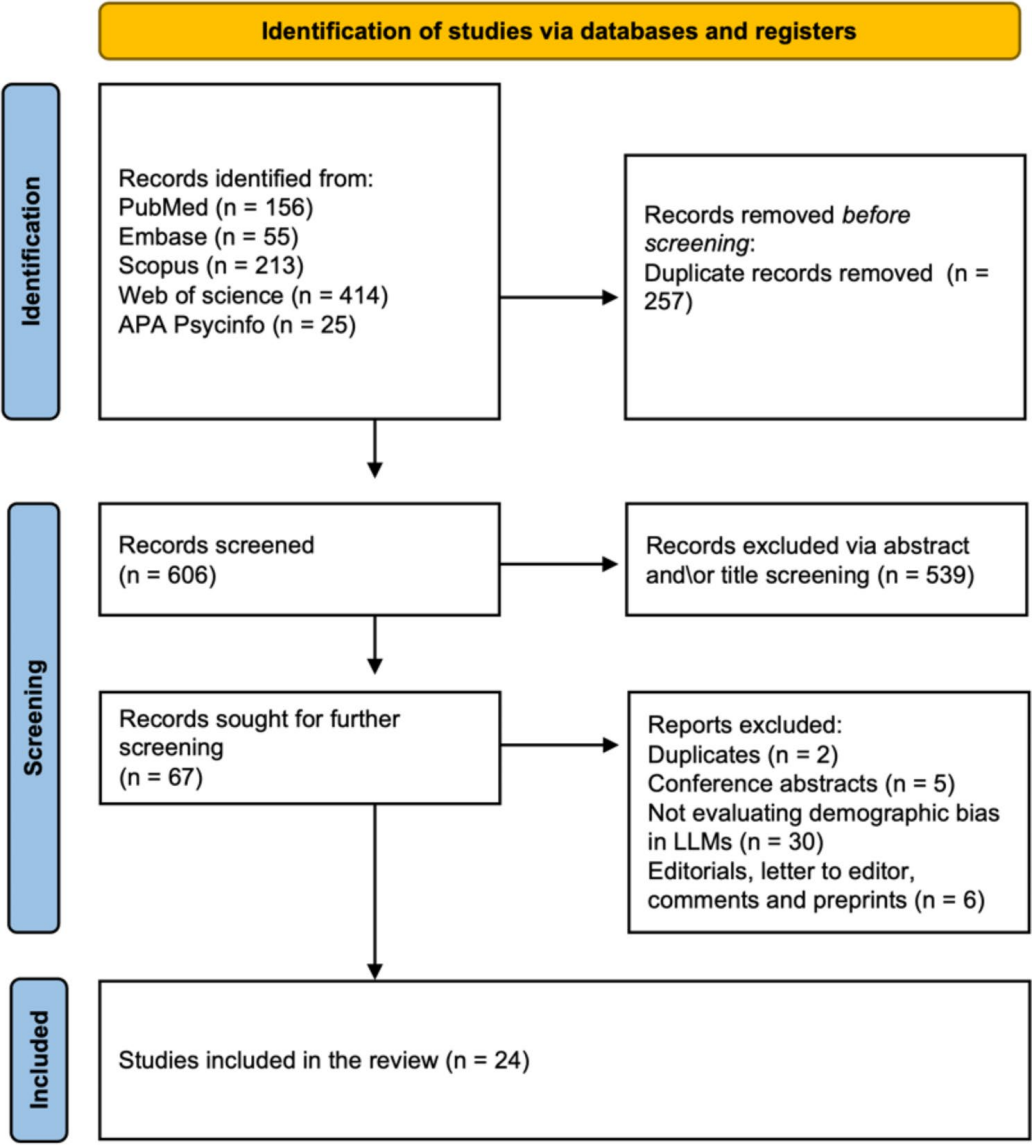
PRISMA flowchart

A total of 863 articles were identified through initial screening. After the removal of 257 duplicates and excluding 539 articles through title and abstract screening, 67 articles underwent full-text review. Ultimately, 24 studies met al.l inclusion criteria [3, 6, 16–37]. A PRISMA flowchart visually represents the screening process in Fig. 1.

### Summary of the included studies

The 24 studies included were published between 2021 and 2024 [3, 6, 16–37], predominantly from the United States, with contributions from other countries including Germany, the Netherlands, Spain, and Turkey (Table 1).

**Table 1:** Summary of the characteristics and results of the included studies

Author et al.	Year	Country	Model Evaluated	Type of Bias Studied	Summary of the results
Elyoseph et al.	2024	Israel/UK	GPT-4, Google Bard	Gender	No discernible gender bias in emotion recognition
Kaplan et al.	2024	USA	GPT-3.5	Gender	Significant gender bias in recommendation letter generation
Bakkum et al.	2024	Netherlands	GPT-3.5	Gender	Gender bias in case generation; proposed mitigation strategy
Bhardwaj et al.	2021	Singapore	BERT	Gender	Significant gender bias in downstream tasks
Shihadeh et al.	2022	USA	GPT-3, InstructGPT	Gender	Substantial “Brilliance Bias” attributing higher achievements to men
Garrido-Muñoz et al.	2023	Spain	Various Spanish LLMs	Gender	Significant gender bias in adjective associations
Srinivasan et al.	2022	USA	VL-BERT	Gender	Gender biases overriding visual evidence in multimodal tasks
Bozdag et al.	2024	Turkey	LegalBERT-Small	Gender	Significant gender bias in medical legal language models
Gross et al.	2023	Ireland	GPT-4	Gender	Perpetuation of gender stereotypes in responses
Lozoya et al.	2023	Australia	GPT-3	Gender	Gen der stereotypes in synthetic mental health data
Cevik et al.	2024	Australia	GPT-3.5, BARD	Gender, racial	Significant gender and skin-tone biases in AI-generated images
Palacios Barea et al.	2023	Netherlands	GPT-3	Gender, racial	Significant biases reflecting social stereotypes
Acerbi et al.	2023	Italy/UK	GPT-3	Gender, social, threat-related	Human-like content biases in information transmission
Doughman et al.	2023	UAE	BERT, DistilBERT	Gender, racial, class, religious	Sexism most prominent; higher bias against females
Smith et al.	2024	USA	GPT-3.5, Claude AI	Racial, ethnic	Biases in student advising recommendations
Amin et al.	2024	USA	GPT-3.5, GPT-4	Racial, ethnic	Bias in simplification of radiology reports based on racial context
Yang et al.	2024	USA	GPT-3.5-turbo, GPT-4	Racial	Significant racial biases in medical report generation
Hanna et al.	2023	USA	GPT-3.5	Racial, ethnic	No significant bias in healthcare-related text generation
Ito et al.	2023	Japan	GPT-4	Racial, ethnic	No significant bias in diagnostic accuracy across racial groups
Xie et al.	2024	USA	Clinical_BERT	Racial, ethnic, gender, socioeconomic	Little intrinsic bias but revealed demographic disparities in outcomes
Zack et al.	2024	USA	GPT-4	Racial, ethnic, gender	Biases in medical diagnosis and treatment recommendations
Andreadis et al.	2024	USA	GPT-4	Racial, ethnic, age, sex	No significant diagnostic bias but age bias in recommendations
Valencia et al.	2024	USA	GPT-3.5, GPT-4.0	Cultural, linguistic	High accuracy and cultural sensitivity; minimal bias
Yeh et al.	2023	Taiwan	GPT-3.5	Age, disability, socioeconomic	Biases when no context provided, mitigated with context

*Gender bias* was the most frequently evaluated type (16 studies), followed by *racial and ethnic bias* (11 studies). Other biases examined included *age*,* disability*,* socioeconomic status*,* and sexual orientation*. The studies evaluated various LLMs, including GPT variants (10 studies), BERT variants (7 studies), and other models like ELECTRA and RoBERTa. Methodologies these studies employed for bias detection and measurement varied widely, including prompt-based testing, corpus analysis, task-specific evaluations, and sentiment analysis. Several studies employed statistical techniques such as log-odds ratios, while others used custom metrics or adapted existing frameworks like the Stereotype Content Model [38] (Table S3 in the supplement provides an in-depth summary of the methodologies of bias detection in the included studies).

Out of 24 studies, 22 (91.7%) identified biases in LLMs. Specifically, 15 of 16 studies (93.7%) reported gender disparities, often reflecting traditional gender roles and stereotypes. Additionally, 10 of 11 studies (90.9%) observed racial or ethnic biases, which typically influenced treatment recommendations, language use, or diagnostic accuracy. Pervasive cultural, age, and intersectional disparities were apparent in all evaluated studies (100% of 3, 2, and 3 studies, respectively), while socioeconomic and language biases were noted in 50% of 2 studies each (Figure S1 in the supplement).

The studies revealed biases across various LLM tasks in healthcare applications. Newer models like GPT showed demographic bias mainly in text generation tasks, such as creating clinical vignettes and discharge instructions. These models also exhibited bias in prediction tasks, including patient outcome forecasting and diagnostic test recommendations, though to a lesser extent. Older models like BERT displayed bias primarily in classification tasks, with responses differing based on patient race and gender.

Regarding mitigation strategies, 7 studies (29%) implemented explicit methods. Of these, 4 used prompt engineering techniques, and 3 applied debiasing algorithms. Six of the seven studies reported reduced disparities in outcomes after implementing mitigation strategies, showcasing improved fairness in medical applications (Figure S1 in the supplement).

### Quality assessment

The quality assessment used two JBI tools: the Critical Appraisal Checklist for Diagnostic Test Accuracy Studies (3 studies) and the Critical Appraisal Checklist for Analytical Cross-Sectional Studies (21 studies) (Tables S1-2 in the supplements). Of the 24 studies evaluated, 8 (33.3%) met all applicable criteria. Across all studies, 177 criteria were met (73.8%), 21 were not met (8.8%), 13 were unclear (5.4%), and 29 were not applicable (12.1%). Studies most often met the JBI tools` criteria related to study design, sample definition, and outcome measurement. Weaknesses included identification and handling of confounding factors, with 7 studies (29.2%) failing to meet or unclear on these criteria. Statistical analysis appropriateness was another concern, with 3 studies (12.5%) not meeting this criterion. The diagnostic accuracy studies generally performed well, meeting most criteria. The cross-sectional studies showed more variability, particularly in addressing confounding factors and statistical analysis.

### Gender bias and mitigation strategies

**Fig. 2:**
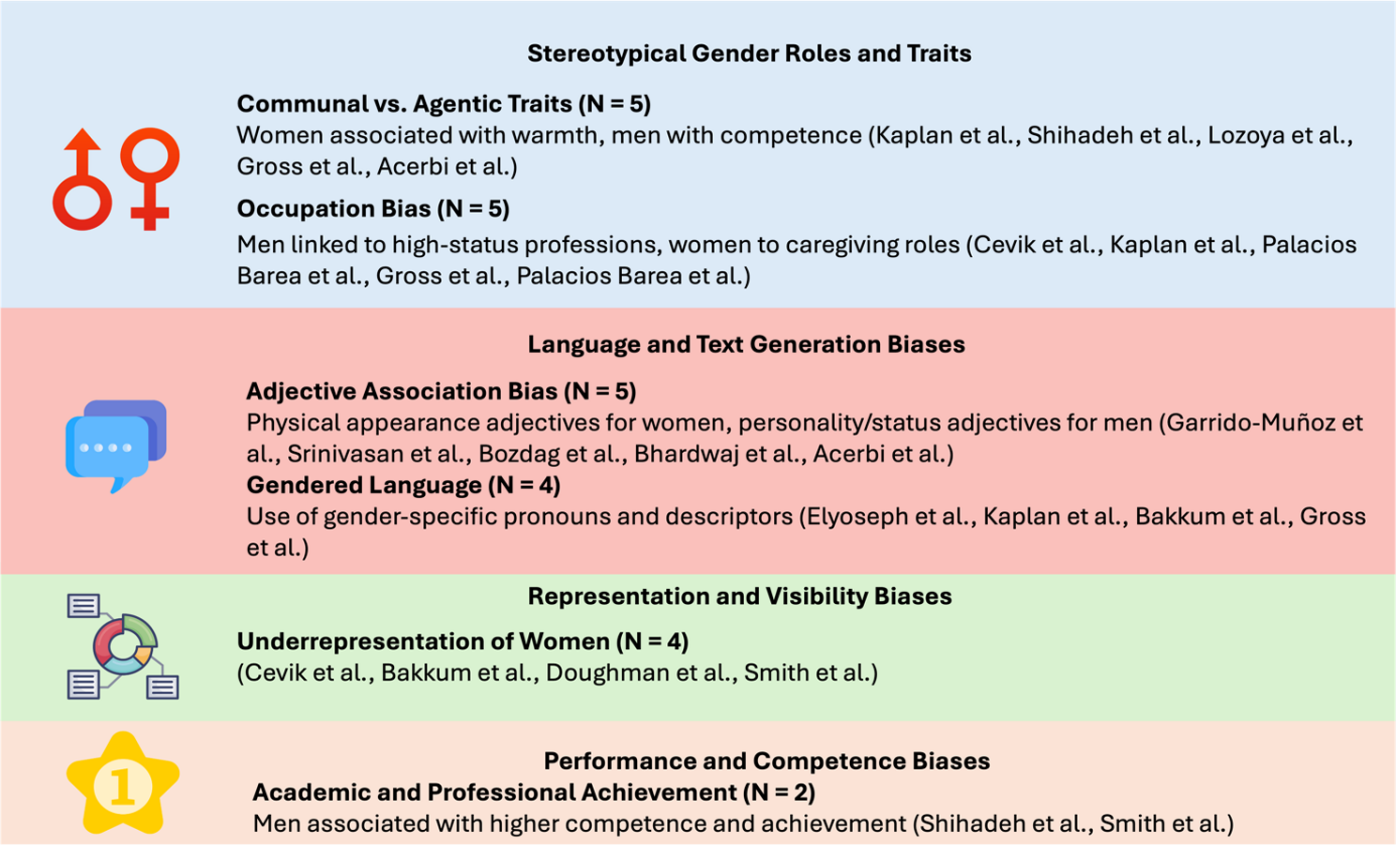
A numeric overall analysis of the detected bias and mitigation strategies. Gender bias manifestations in LLMs

Gender bias was evaluated in 16 studies across various LLMs and different applications, including GPT variants and BERT variants, with 93.7% confirming its presence. For instance, Kaplan et al., Bhardwaj et al., and Bozdag et al. observed gender bias in text generation tasks [29, 32, 36]. Kaplan et al. found that GPT-3.5 recommendation letters for men included more agentic terms, which describe qualities of assertiveness, independence, and achievement, significantly more than for women who were described using communal language [36]. Bhardwaj et al. noted BERT assigned more competence-related traits to male-generated text and more warmth-related traits to female-generated text [29]. Bozdag et al. reported gender bias in medical legal contextualized language models affected task performance [32] (Fig. 2).

Bias was also noted in visual tasks. Srinivasan et al. and Gross et al. identified gender stereotypes in visual-linguistic tasks and general responses [3, 37]. Srinivasan et al. found VL-BERT overrode visual evidence in favor of learned gender biases [37], while Gross et al. reported that GPT reinforced traditional gender roles in its responses [3].

Garrido-Muñoz et al. and Lozoya et al. examined gender bias in non-English contexts [28, 31]. Garrido-Muñoz et al. found Spanish language models showed strong bias in describing females with body-related adjectives and males with behavior-related adjectives [31]. Lozoya et al. observed gender stereotypes in synthetic mental health data generated by GPT-3 [28].

Shihadeh et al., Palacios Barea et al., and Acerbi et al. explored specific aspects of gender bias [20, 21, 30]. Shihadeh et al. found evidence of “Brilliance Bias” in GPT-3 and InstructGPT, attributing higher achievements to men [21]. Palacios Barea et al. observed GPT-3 reproduced social stereotypes related to gender [20]. Acerbi et al. noted GPT-3 exhibited human-like gender biases in information transmission [30].

On the other hand, Elyoseph et al. found no discernible gender bias in GPT-4’s emotion recognition tasks, contrasting with other studies’ findings [22].Valencia et al. reported that prompt engineering could enhance cultural sensitivity in medical translations using GPT-3.5 and GPT-4.0 [16]. Similarly, Bakkum et al. proposed a similar prompt engineering method to reduce bias in legal language models while maintaining performance [35].

### Racial and ethnic bias

**Fig. 3:**
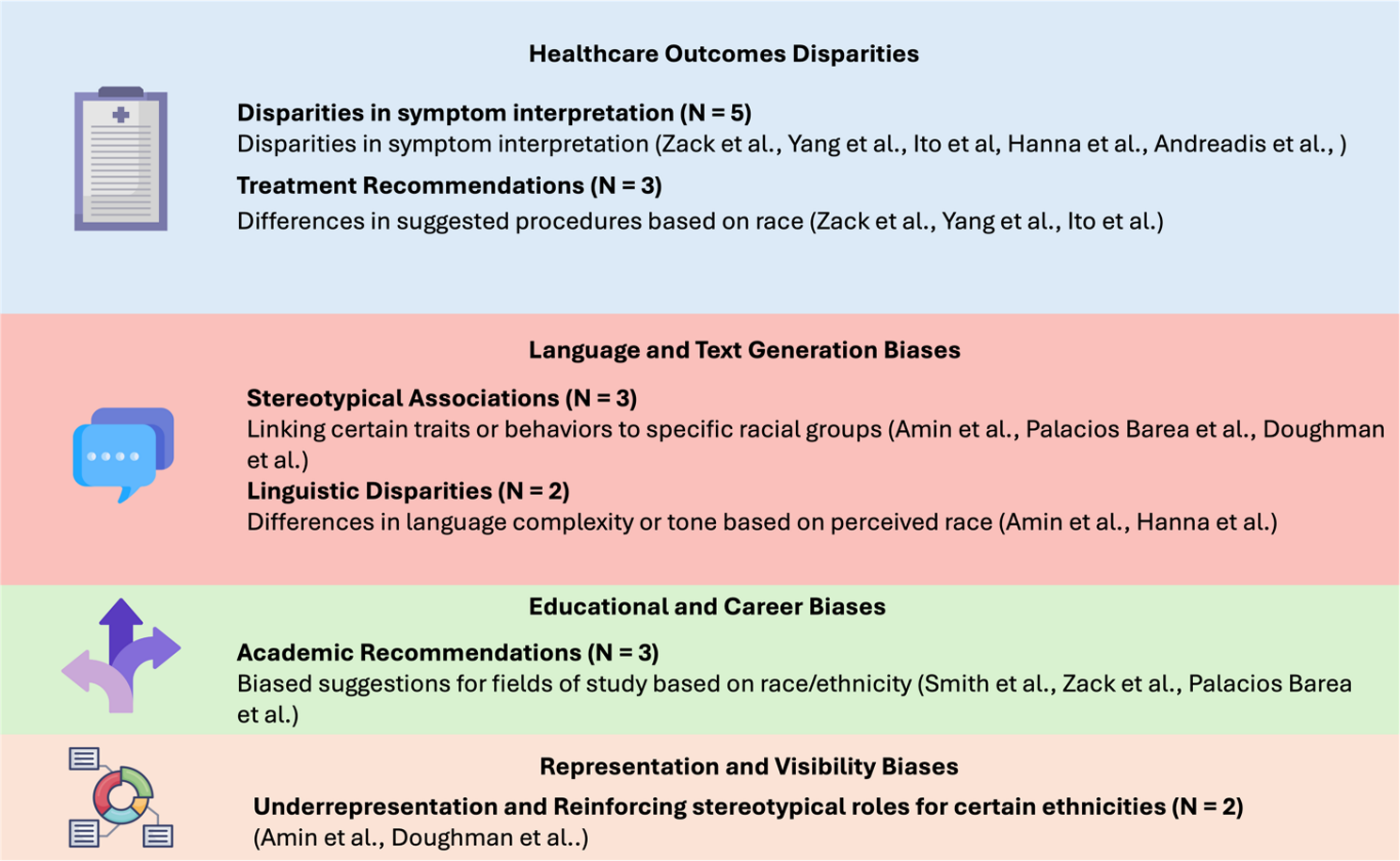
Racial and ethnic biases manifestations in LLMs

Racial and ethnic biases were examined in 11 studies across several applications. Yang et al. found GPT-3.5-turbo exhibited biases in medical report generation across racial groups, including fabricated patient histories and racially skewed diagnoses [6]. Zack et al. reported that GPT-4 showed disparities in recommending advanced imaging, with lower rates of recommendations for patients from underrepresented racial groups compared to those of European descent [18]. In a similar manner, Smith et al. found biases in student advising recommendations when examining GPT-3.5 and Claude AI’s responses to lists of names associated with different racial/ethnic groups [27] (Fig. 3).

Amin et al. observed bias in GPT’s simplification of radiology reports based on racial context, finding statistically significant differences in reading grade levels between racial contexts for both GPT-3.5 and GPT-4 [25]. Conversely, some studies found limited or no evidence of racial bias. Xie et al. observed little intrinsic bias in ClinicalBERT but revealed demographic disparities in outcomes when applied to real-world data [23]. Hanna et al. found no significant differences in polarity and subjectivity across races/ethnicities in GPT’s healthcare-related text generation [17]. Similarly, Ito et al. reported no significant difference in GPT-4’s diagnostic accuracy across racial and ethnic groups when compared to human physicians [34]. Andreadis et al. also reported no significant racial diagnostic bias with GPT-4, although they noted an age-related bias in recommendations [33].

### Other biases

Yeh et al. (2023) conducted a study using GPT-3.5 to examine biases related to age, disability, socioeconomic status, and sexual orientation [24]. The study found that GPT-3.5 exhibited biases across these demographic factors when prompts lacked context [24].

Andreadis et al. observed age-related bias in GPT’s urgent care recommendations, which were presented more frequently to older individuals [33]. Xie et al. found socioeconomic disparities in LLM-extracted seizure outcomes, with patients having public insurance and those from lower-income zip codes showing worse outcomes [23]. Doughman et al. (2023) conducted a study examining multiple types of bias in BERT and DistilBERT models, including gender, racial, class, and religious biases [26]. Their research revealed that sexism was the most prominent form of bias, with a notably higher bias against females. The study found that sexist sentences had the highest match rate, with BERT showing around 24% and DistilBERT showing 16% for sexist content. (Table S4 in the supplement lists some specific examples of different biases from the included studies).

Valencia et al. studied a novel mitigation strategy for bias in language models. They compared GPT translations of kidney transplantation FAQs from English to Spanish against human translations. The researchers used prompt engineering to tailor the translations for the Hispanic community. GPT’s translations showed higher accuracy and cultural sensitivity than human translations. The study found minimal bias in the GPT-generated translations [16] (Table 2).

**Table 2:** Mitigation strategies reported

Author et al.	Year	Country	Model Evaluated	Type of Bias Studied	Mitigation Method	Mitigation Results
Bakkum et al.	2024	Netherlands	GPT 3.5	Gender Bias	**Prompt Engineering**: Iterative Prompt Optimization, Segmented Prompting	Enhanced diversity in medical vignettes; improved inclusivity.
Yeh et al.	2023	Taiwan	GPT-3.5-turbo	Multiple Societal Biases	**Prompt Engineering**: Contextualization and Disambiguation Techniques	Reduced bias through detailed prompts and disambiguation.
Palacios Barea et al.	2023	Netherlands	GPT-3	Gender, Racial Bias	**Prompt Engineering**: Thematic Prompts	Identified and reduced biases in gender and racial representation.
Andreadis et al.	2024	USA	GPT-4	Age, Gender, Racial Bias	**Prompt Engineering**: Demographic Tailoring	Found potential age bias in urgent care recommendations.
Bhardwaj et al.	2021	Singapore	BERT	Gender Bias	**Debiasing Algorithm**: Gender Debiasing Algorithm using PCA	Significantly reduced gender bias in emotion prediction tasks.
Bozdag et al.	2024	Turkey	LegalBERT-Small	Gender Bias	**Debiasing Algorithm**: Legal-Context-Debias (LCD)	Reduced gender bias in legal text while maintaining performance.
Doughman et al.	2023	UAE	DistilBERT	Sexism, Multiple Bias	**Debiasing Algorithm**: Context-Debias Algorithm	Reduced biased predictions in masked language models.

*Abbreviations: PCA: Principal Component Analysis| LCD: Legal-Context-Debias

## Discussion

This systematic review reveals pervasive demographic biases in medical LLMs, with gender and racial/ethnic biases being particularly common. Some studies attempted to mitigate these biases, as prompt engineering and debiasing algorithms showed promise. These findings underscore an important ethical challenge in deploying LLMs for healthcare. They also emphasize the need for rigorous testing and the development of validated mitigation strategies before integrating LLMs into clinical practice.

The reviewed studies employed a range of metrics to quantify bias in large language models, including accuracy scores (0-100%), probability indices (-1 to + 1), and representation percentages (0-100%). Cevik et al.‘s findings on DALL-E2’s image generation demonstrate how AI can perpetuate gender stereotypes in professional roles, potentially influencing societal perceptions of medical professionals [19]. In another interesting and quantifiable record, Yang et al. found GPT-3.5-turbo predicted lower death rates for White patients (56.54%) compared to other racial groups (up to 62.25% for Black patients), suggesting potential racial bias in medical prognosis [6]. Importantly, Garrido-Muñoz et al.‘s work on Spanish language models shows that these biases are not limited to English-language AI, suggesting a widespread issue that crosses linguistic boundaries [31].

The prevalence of these biases across different models and applications highlights ongoing challenges in LLM development. Despite advances in model architecture and training, AI systems continue to reflect possible societal biases. Models like GPT-4 [39], released in March 2023, still produce ethnic, racial, and gender biases. These biases appear mainly in written output text, but also affect prognosis predictions and recommendations for treatments and management protocols [18]. This persistence suggests that addressing bias requires both technical solutions and examination of the data and societal contexts in which these models are trained on, and that use of LLMs should be carefully considered to avoid perpetuating those biases.

Gender bias emerged as the most frequently observed and studied form of bias among the reviewed articles. Many studies found gender bias in tasks such as recommendation letter generation [36], medical case generation [35], and diagnostic reasoning [37]. Several investigations noted more specific issues like “Brilliance Bias,” where higher achievements are attributed to men [21], while others reported more subtle patterns in adjective associations [31]. Yet, most studies focused on cisgender men and women, with almost no current work addressing non-binary gender identities. This narrow focus underscores the need to broaden our understanding of gender bias and develop more inclusive strategies for mitigating it in medical LLMs.

Although some medical distinctions between sexes are clinically warranted, the findings in our review suggest that many LLM-generated recommendations may not be rooted in valid physiological variability. Instead, they often appear to reflect biases unrelated to biology or evidence-based practice. Furthermore, because these evaluations primarily focused on gender identity, rather than sex, it remains unclear whether legitimate sex-based variations were captured at all.

Mitigation strategies were explored in several studies, though less prominently than bias detection methods, and quantitative data on their effectiveness remains limited. The lack of standardized metrics for measuring bias reduction complicates comparisons across studies. These findings underscore the pervasive nature of demographic biases in LLMs and emphasize the need for more robust, quantifiable mitigation strategies.

Approaches for bias mitigation included prompt engineering and specialized debiasing algorithms, and more importantly, continued human oversight. For example, Valencia et al. demonstrated that fine-tuning AI chatbots improved cultural sensitivity in medical translations. These chatbots were optimized for translation accuracy and cultural relevance, focusing on nuances specific to the Hispanic community [16]. Interestingly, Valencia et al. concluded that fine-tuned GPT-3.5 and GPT-4 have the potential to promote health equity by enhancing access to essential kidney transplant information in Spanish. GPT-4 was found to be more sensitive and ethnically accurate than GPT-3.5, supporting the development of more advanced and culturally sensitive LLMs [16]. Additionally, Bakkum et al. proposed a method, using iterative prompt optimization and segmented prompting to reduce gender bias in medical legal language models [35]. Moreover, Bhardwaj et al. reported a 63.9% reduction in gender bias metrics for BERT models, using debiasing algorithms for BERT [29]. These strategies show promise, but their effectiveness varies across bias types and application contexts and require further validation on large datasets and models [40].

The potential of LLMs to mitigate bias shows promise but remains complex. Some studies indicate that advanced LLMs can reduce biases in human-generated text [41–43]. However, their rapid development and widespread adoption across various fields present ongoing challenges. The models’ training data, both current and historical, contains inherent biases that will likely persist in the near future [24]. We propose that developing validated bias mitigation methods for human data could positively impact the creation of less biased models. These methods could be applied to the same data used for further training and development, potentially reducing bias in future LLMs. This requires robust evaluation in real-world medical scenarios. Studies should assess how these mitigation approaches affect model accuracy and efficiency, especially for decision-making. One proposed approach is removing references to race, gender, or other potentially sensitive categories [29]. However, this could have unintended consequences in clinical settings where sex-based distinctions are medically relevant. Future research should carefully balance bias reduction with maintaining clinically important information.

Yet, LLMs hold much promise for medical integration, streamlining tasks, and potentially saving valuable time and resources [2]. Recent evidence shows that these models have established diagnostic capabilities and can combine different types of outputs in multimodal LLMs for diagnosis, treatment, and decision-making [44–46]. Although our review highlights how these LLMs could perpetuate biases at their current stage, we believe this underscores the need for more effective mitigation efforts. Addressing these biases will enable safer and more equitable integration of medical AI in everyday clinical practice.

Biases in LLM-generated recommendations can have tangible consequences for clinical outcomes. For instance, over-triaging marginalized populations could strain already limited healthcare resources and potentially result in unnecessary interventions, contributing to an estimated $760–935 billion in annual waste in the U.S. healthcare system [44]. At the same time, under-triaging other groups may delay necessary care, affecting their health outcomes. Biases favoring advanced diagnostics for high-income patients could further widen existing gaps in diagnostics for low-income individuals [46]. In some cases, biases might lead to misdiagnosis (e.g., labeling symptoms as psychological), which diverts attention from critical medical issues [45].

Current research on demographic biases in LLMs has limitations. Few studies address biases related to sexual orientation, non-binary gender identities, and intersectional identities. The focus on binary gender categories fails to capture the full spectrum of gender identities [47]. Additionally, the geographical concentration of studies in Western countries limits our understanding of biases in diverse cultural contexts [48]. Tailoring LLMs to specific countries and cultures may help address these gaps by incorporating local norms, languages, healthcare practices, and societal values. To advance this field, future research should prioritize evaluating a wider range of demographic factors and intersectional analyses. Developing robust, context-aware mitigation strategies is essential, as is establishing ethical guidelines for LLM deployment. Researchers should investigate biases in non-Western cultural contexts and explore the impact of different training data sets on bias formation, including studies on non-English speakers. In addition, more models should be evaluated, as the current literature mainly focuses on GPT models.

In conclusion, Biases are observed in LLMs across various medical domains. While bias detection is improving, effective mitigation strategies are still developing. As LLMs increasingly influence critical decisions, addressing these biases and their resultant disparities is essential for ensuring fair AI systems. Future research should focus on a wider range of demographic factors, intersectional analyses, and non-Western cultural contexts.

## Electronic supplementary material

Below is the link to the electronic supplementary material.

Supplementary Material 1

## Data Availability

No datasets were generated or analysed during the current study.
