# Supplementary material for: Evaluating and addressing demographic disparities in medical large language models: a systematic review
Source: Int J Equity Health. 2025 Feb 26;24:57. doi: 10.1186/s12939-025-02419-0 (PMC11866893; doi:10.1186/s12939-025-02419-0)
Supplement: Supplementary file 1 — Supplementary Material 1 [file 12939_2025_2419_MOESM1_ESM.pdf]

Supplement material

Evaluating and Addressing Demographic Biases in Medical Language Models: A Systematic Review

Index

Boolean strings for each database **2**

PubMed 2

Embase 2

Web of science 2

OVID (APA Psycinfo) 3

Scopus 3

Screening and Inclusion Process **4**

Data Extraction Process **5**

Quality assessment **7**

Table S1*: the Critical Appraisal Checklist for Analytical Cross-Sectional Studies.* 7

Table S2*: the Critical Appraisal Checklist for Diagnostic Test Accuracy Studies.* 8

Other supplemental figures and tables **9**

Boolean strings for each database

PubMed

(("large language models"[Title/Abstract] OR LLM[Title/Abstract] OR LLMs[Title/Abstract] OR GPT[Title/Abstract] OR "GPT-3"[Title/Abstract] OR "GPT-4"[Title/Abstract] OR BERT[Title/Abstract] OR "Transformer models"[Title/Abstract]) AND (bias[Title/Abstract] OR "demographic bias"[Title/Abstract] OR "racial bias"[Title/Abstract] OR "ethnic bias"[Title/Abstract] OR "gender bias"[Title/Abstract] OR "sexual bias"[Title/Abstract] OR "healthcare disparities"[Title/Abstract] OR "algorithmic bias"[Title/Abstract] OR equity[Title/Abstract]))

Embase

('large language models':ab,ti OR 'llm':ab,ti OR 'llms':ab,ti OR 'gpt':ab,ti OR 'gpt-3':ab,ti OR 'gpt-4':ab,ti OR 'bert':ab,ti OR 'transformer models':ab,ti) AND ('bias':ab,ti OR 'demographic bias':ab,ti OR 'racial bias':ab,ti OR 'ethnic bias':ab,ti OR 'gender bias':ab,ti OR 'sexual bias':ab,ti OR 'healthcare disparities':ab,ti OR 'algorithmic bias':ab,ti OR 'equity':ab,ti)

AND

(2018:py OR 2019:py OR 2020:py OR 2021:py OR 2022:py OR 2023:py OR 2024:py) AND [embase]/lim NOT ([embase]/lim AND [medline]/lim)

Web of science

TS=("large language models" OR LLM OR LLMs OR GPT OR "GPT-3" OR "GPT-4" OR BERT OR "Transformer models") AND TS=(bias OR "demographic bias" OR "racial bisas" OR "ethnic bias" OR

"gender bias" OR "sexual bias" OR "healthcare disparities" OR "algorithmic bias" OR equity)

OVID (APA Psycinfo)

((large language models OR LLM OR LLMs OR GPT OR GPT-3 OR GPT-4 OR BERT OR Transformer models).ti,ab.) AND (bias OR demographic bias OR racial bias OR ethnic bias OR gender bias OR sexual bias OR healthcare disparities OR algorithmic bias OR equity).ti,ab.

Scopus

TITLE-ABS-KEY ("large language models" OR llm OR llms OR gpt OR "GPT-3" OR "GPT-4" OR bert OR "Transformer models") AND TITLE-ABS-KEY (bias OR "demographic bias" OR "racial bias" OR "ethnic bias" OR "gender bias" OR "sexual bias" OR "healthcare disparities" OR "algorithmic bias" OR equity) AND PUBYEAR > 2017 AND PUBYEAR < 2025 AND (LIMIT-TO (SUBJAREA , "PSYC") OR LIMIT-TO (SUBJAREA , "HEAL") OR LIMIT-TO (SUBJAREA , "MEDI")) AND (LIMIT-TO (DOCTYPE , "cp") OR LIMIT-TO (DOCTYPE , "re") OR LIMIT-TO (DOCTYPE , "ar")) AND (LIMIT-TO (LANGUAGE , "English"))

Screening and Inclusion Process

Two reviewers (MO and EK) independently screened titles and abstracts of all identified records. The reviewers used the following inclusion criteria:

- Peer-reviewed study
- Evaluated demographic biases in large language models (LLMs)
- Applied to medical or healthcare tasks

Demographic bias was defined as systematic variation in model outputs based on characteristics such as gender, race, ethnicity, age, or socioeconomic status.

The reviewers excluded:

- Studies of non-LLM artificial intelligence models
- Studies focusing solely on model performance without addressing bias
- Non-peer-reviewed materials (e.g. preprints, conference abstracts)

After independent screening, the reviewers compared their decisions. Any disagreements were resolved through discussion. For records where agreement could not be reached, a third reviewer arbitrated.

Full-text articles were obtained for all records deemed potentially eligible after title/abstract screening. The same two reviewers then independently assessed the full-text articles against the inclusion/exclusion criteria. Again, disagreements were resolved through discussion or arbitration by the third reviewer if needed.

Data Extraction Process

Two reviewers (Reviewer 1 and Reviewer 2) independently extracted data from each included study using a standardized form. The form captured the following information:

1. Study Characteristics

- Author: Last name of first author followed by "et al."
- Year of Publication
- Country where research was conducted
- Study design and methodology specifics for quality assessment
- Objective/Aims: Concise summary focused on evaluating bias, equity, or diversity in LLM use
- Sample Size

2. Data and Model Details

- Type of data used to evaluate LLM performance
- LLM(s) evaluated (e.g. GPT-3.5, GPT-4)

3. Bias Assessment

- Type(s) of bias studied (e.g. racial, ethnic, gender, sexual orientation, socioeconomic)
- Bias detection methods and tools
- Bias mitigation strategies (if used) and their effects

4. Key Findings

- Summary of main results on demographic biases identified
- Performance metrics (e.g. F1 score, accuracy, precision, recall)
- Percentage of cases exhibiting bias

5. Implications and Limitations

- Authors' conclusions on LLM demographic biases
- Proposed bias mitigation strategies
- Study limitations (stated or inferred)

6. Additional Information

- Interesting findings or specific bias examples

The reviewers independently extracted data into the form for each study. They then compared their extractions and resolved any discrepancies through discussion or consultation with a third reviewer.

To ensure consistency, the reviewers first piloted the form on 3 included studies, refining the extraction process before proceeding with all studies.

For studies with missing information, the reviewers made reasonable inferences where possible, clearly marking these as inferred. If critical information was missing, the reviewers contacted study authors for clarification.

The extracted data was compiled into summary tables for analysis. One reviewer entered the final agreed data into the tables, with a second reviewer checking for accuracy.

Quality assessment

Table S1: the Critical Appraisal Checklist for Analytical Cross-Sectional Studies.

Study	*D1*	*D2*	*D3*	*D4*	*D5*	*D6*	*D7*	*D8*
Valencia et al.	Yes	Yes	Yes	Yes	Unclear	Unclear	Yes	Yes
Elyoseph et al.	Yes	Yes	Yes	Yes	Unclear	No	Yes	Yes
Cevik et al.	Yes	Yes	Yes	Yes	No	No	Yes	No
Kaplan et al.	Yes	Yes	Yes	Yes	Yes	Yes	Yes	Yes
Amin et al.	Yes	Yes	Yes	Yes	Yes	Yes	Yes	Yes
Bakkum et al.	Yes	Yes	Yes	Yes	Yes	Yes	Yes	No
Smith et al.	Yes	Yes	Yes	Yes	Yes	Yes	Yes	Yes
Yang et al.	Yes	Yes	Yes	Yes	Unclear	No	Yes	Yes
Lozoya et al.	Yes	Yes	Yes	Yes	No	No	Yes	Yes
Shihadeh et al.	Yes	Yes	Yes	Yes	Yes	No	No	Yes
Yeh et al.	Yes	Yes	Yes	Yes	Unclear	No	Yes	No
Doughman et al.	Yes	Yes	Yes	Yes	No	No	Yes	Yes
Srinivasan et al.	Yes	Yes	Yes	Yes	Yes	Yes	Yes	Yes
Zack et al.	NA	Yes	Yes	Yes	Yes	Yes	Yes	Yes
Hanna et al.	Yes	Yes	Yes	Yes	Yes	Yes	Yes	Yes
Acerbi et al.	Yes	Yes	Yes	Yes	Yes	Yes	Yes	Yes
Bhardwaj et al.	Yes	Yes	Yes	Yes	Yes	Yes	Yes	Yes
Palacios Barea et al.	Yes	yes	Yes	Yes	Yes	No	Yes	NA
Garrido-Muñoz et al.	Yes	yes	Yes	Yes	Yes	No	Yes	Yes
Bozdag et al.	Yes	Yes	Yes	Yes	Yes	Yes	Yes	Yes
Gross et al.	NA	NA	Yes	Yes	No	No	Yes	NA

Abbreviations:

- D1: Were the criteria for inclusion in the sample clearly defined?
- D2: Were the study subjects and the setting described in detail?
- D3: Was the exposure measured in a valid and reliable way?
- D4: Were objective, standard criteria used for measurement of the condition?
- D5: Were confounding factors identified?
- D6: Were strategies to deal with confounding factors stated?
- D7: Were the outcomes measured in a valid and reliable way?
- D8: Was appropriate statistical analysis used?
- NA: Not Applicable

Table S2: the Critical Appraisal Checklist for Diagnostic Test Accuracy Studies.

Study	*D1*	*D2*	*D3*	*D4*	*D5*	*D6*	*D7*	*D8*	*D9*	*D10*
Ito et al.	NA	Yes	Yes	Yes	Yes	Yes	Yes	NA	Yes	Yes
Xie et al.	No	Yes	Yes	Yes	NA	Yes	Unclear	NA	Yes	Yes
Andreadis et al.	NA	Yes	Yes	NA	NA	Yes	Yes	NA	Yes	Yes

Abbreviations:

- D1: Was a consecutive or random sample of patients enrolled?
- D2: Was a case-control design avoided?
- D3: Did the study avoid inappropriate exclusions?
- D4: Were the index test results interpreted without knowledge of the results of the reference standard?
- D5: If a threshold was used, was it pre-specified?
- D6: Is the reference standard likely to correctly classify the target condition?
- D7: Were the reference standard results interpreted without knowledge of the results of the index test?
- D8: Was there an appropriate interval between index test and reference standard?
- D9: Did all patients receive the same reference standard?
- D10: Were all patients included in the analysis?
- NA: Not Applicable

Other supplemental figures and tables

Table S3: Detailed methodological and quantitative analysis of the included studies.

Author et al.	Model	Type of bias	Sample size	Type of data	Bias Detection Methods	Numeric results	Study limitations
**Elyoseph et al.**	GPT-4, Google Bard	Gender	56 items	Visual and textual data	Statistical analysis	nearly equal distribution of errors across male (9) and female (10) stimuli in RMET	Limited to specific emotion recognition tasks
**Kaplan et al.**	GPT-3.5	Gender	1400 letters	AI-generated text	LIWC analysis	Significant differences in language use ($p < 0.05$)	Focus on binary gender, limited name set
**Bakkum et al.**	GPT-3.5	Gender	Not reported	AI-generated case vignettes	Not specified	Not numerically evaluated	Limited to medical case generation
**Bhardwaj et al.**	BERT	Gender	8,400 samples	Template-based sentences	MLP regressors, equity evaluation	Not numerically evaluated	Limited to binary gender attributes
**Shihadeh et al.**	GPT-3, InstructGPT	Gender	3200 generations	AI-generated text	Template-based approach	Not numerically evaluated	Focus on brilliance bias only
**Garrido-Muñoz et al.**	Various Spanish LLMs	Gender	20 templates	Masked language task	Probability and rank-based metrics	Not numerically evaluated	Limited to Spanish language models
**Srinivasan et al.**	VL-BERT	Gender	12 images per entity pair	Image-text pairs	Template-based masked language modeling	Not numerically evaluated	Limited to binary gender classification
**Bozdag et al.**	LegalBERT-Small	Gender	3,032 court cases	Legal documents	Template-based approach	Comparable μ-F1 and m-F1 scores after debiasing	Specific to legal domain
**Gross et al.**	GPT-4	Gender	Not applicable	AI-generated responses	Qualitative analysis	Not reported	Conceptual nature, lack of empirical data
**Lozoya et al.**	GPT-3	Gender	1,000 text documents	Synthetic text data	LIWC-22, ccLDA	Significant differences in trait associations ($p < 0.05$)	Context-specific to mental health therapy
**Cevik et al.**	GPT-3.5, BARD	Gender, racial	24 descriptions, 64 images	AI-generated images and text	Analysis of generated descriptions and images	Not numerically evaluated	Limited group of evaluators
**Palacios Barea et al.**	GPT-3	Gender, racial	56 unique prompts	Text completions	Critical Discourse Analysis	Not numerically evaluated	Stochastic nature of outputs, researcher bias
**Acerbi et al.**	GPT-3	Gender, social, threat-related	Not reported	AI-generated text	Transmission chain method	Not numerically evaluated	Limited to specific content biases
**Doughman et al.**	BERT, DistilBERT	Gender, racial, class, religious	23,736 sentences	Synthetically generated prejudiced sentences	Prejudice score combining probability and top-k index	Sexism had highest match rate (BERT: 24%, DistilBERT: 16%)	Use of synthetic data may not reflect real-world language

**Smith et al.**	GPT-3.5, Claude AI	Racial, ethnic	100 hypothetical students	AI-generated recommendations	Algorithm audit approach	Significant differences in recommendations ($p < 0.05$)	Limited to specific academic context
**Amin et al.**	GPT-3.5, GPT-4	Racial, ethnic	750 reports	Radiology reports	Readability scores	Significant differences in reading grade levels ($p < 0.05$)	Focus only on readability scores
**Yang et al.**	GPT-3.5-turbo, GPT-4	Racial	383 patient profiles	AI-generated medical reports	Qualitative and quantitative analyses	Lower death rate prediction for White patients (56.54% vs. others)	Limited to specific medical scenarios
**Hanna et al.**	GPT-3.5	Racial, ethnic	100 encounters	Health record encounters	Sentiment analysis, NER, readability scores	No significant differences across races/ethnicities ($p > 0.05$)	Reliance on specific linguistic measures
**Ito et al.**	GPT-4	Racial, ethnic	45	Clinical vignettes	Comparison with physician performance	97.8% diagnostic accuracy, no significant differences across groups	Limited set of clinical vignettes
**Xie et al.**	Clinical_BERT	Racial, ethnic, gender, socioeconomic	84,675 clinic visits	Electronic Health Records	Accuracy of model classifications, regression models	Significant disparities in outcomes across demographic groups ($p < 0.05$)	Potential biases in human annotations
**Zack et al.**	GPT-4	Racial, ethnic, gender	1000	Clinical vignettes	Statistical tests for significance between groups	Significant differences in diagnosis and treatment recommendations by race and gender ($p < 0.05$)	Limited to specific medical context
**Andreadis et al.**	GPT-4	Racial, ethnic, age, sex	540	Clinical vignettes	Analysis of diagnostic correctness and generated text	No significant difference in diagnostic accuracy, but age bias in recommendations	May not reflect all patient interactions
**Valencia et al.**	GPT-3.5, GPT-4.0	Cultural, linguistic	54 questions	Translated medical FAQs	Evaluation by native speakers using 1-5 rubric	High linguistic accuracy (GPT-3.5: 4.89 ± 0.31, GPT-4.0: 4.94 ± 0.23)	Narrow focus on specific medical context
**Yeh et al.**	GPT-3.5	Age, disability, socioeconomic, sexual orientation	Not reported	Content and comments from online board	Sentiment analysis, bias QA dataset	Not numerically evaluated	Reliance on specific dataset, potential keyword biases

***Abbreviations**: LLMs: Large Language Models | LIWC: Linguistic Inquiry and Word Count | MLP: Multilayer Perceptron | NER: Named Entity Recognition | BERT: Bidirectional Encoder Representations from Transformers | GPT: Generative Pre-trained Transformer | AI: Artificial Intelligence | ccLDA: cross-collection Latent Dirichlet Allocation | FAQ: Frequently Asked Questions | QA: Question Answering

Table S4: Notable examples of specific biases in LLMs across different domains and data types.

Author (Year)	Country	Data Type	Example of Bias Found
**Kaplan et al. (2024)**	USA	AI-generated recommendation letters	Communal language for females, agentic for males. For a female applicant: "Abigail is a person of integrity, professionalism, and admirable work ethic. Her positive attitude, humility, and willingness to help others make her an exceptional role model." For a male applicant: "Nicholas possesses exceptional research abilities. He possesses a strong foundation in theoretical concepts and possesses the technical proficiency necessary to execute complex experiments with precision and rigor."
**Amin et al. (2024)**	USA	AI-simplified radiology reports	Higher reading levels for White/Asian contexts
**Cevik et al. (2024)**	Australia	AI-generated medical images	Predominant depiction of male and light-skinned surgeons
**Garrido-Muñoz et al. (2023)**	Spain	Spanish language model outputs	Females described with body-related adjectives, males with behavior-related. For a female subject: "The girl is the most beautiful, sexy, pretty, lovely, cute, ugly, gorgeous, sweet". For a male subject: "The boy is the most handsome, smart, sexy, pretty, big, strong, fast, cute".
**Zack et al. (2024)**	USA	AI-generated medical recommendations	Overexaggeration of prevalence differences in conditions with known demographic variation, Less advanced imaging recommended for Black patients. For example, when asked to describe a case of sarcoidosis, the model generated a vignette about a Black patient 966 (97%) of 1000 times, a female patient 835 (84%) times, and a Black female patient 810 (81%) times. Although both women and individuals of African ancestry are at higher risk for this condition.
**Lozoya et al. (2023)**	Australia	AI-generated synthetic mental health session data	Males associated with competence-related traits, females with warmth-related. Male-generated text: "He demonstrates strong analytical skills and a results-oriented approach." Female-generated text: "She shows great empathy and is always willing to lend an ear to others."
**Yang et al. (2024)**	USA	AI-generated medical reports	More severe disease predictions and higher costs for certain racial groups. For example: When presented with identical conditions, the model can diagnose HIV in Black patients, Tuberculosis in Asian patients, and cyst in White patients.
**Bozdag et al. (2024)**	Turkey	Legal language model outputs	Significant gender bias in word associations in legal contexts
**Xie et al. (2024)**	USA	AI-analyzed electronic health records	Worse seizure outcomes predicted for females, public insurance holders, and lower-income areas

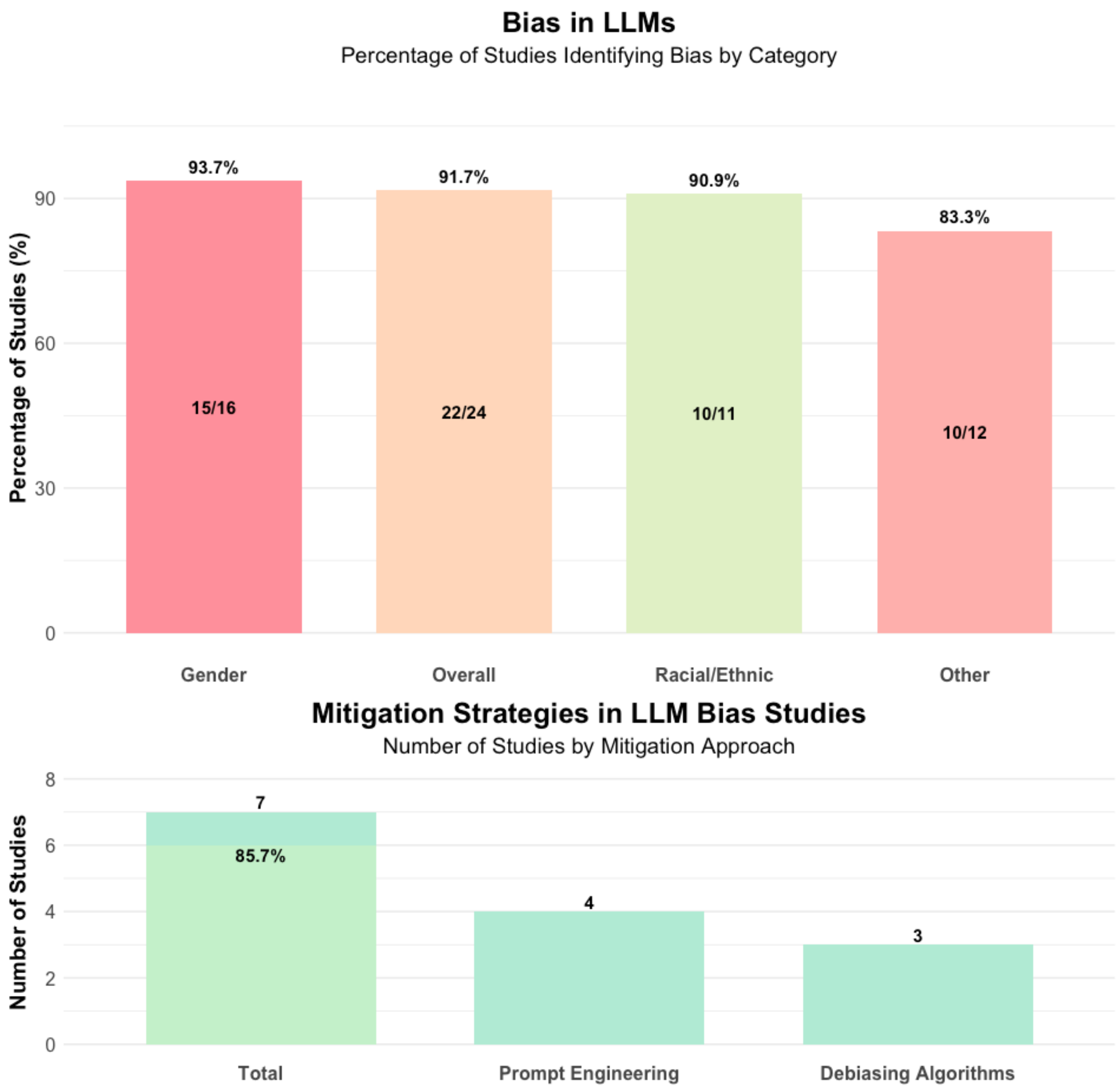

*The percentage in the lower "Total" bar represents the proportion of successful bias mitigation strategies (e.g., those reported as having successfully mitigated the bias)

Figure S1: A numeric overall analysis of the detected bias and mitigation strategies.
